# From a crisis to an opportunity: Eight insights for doing science in the COVID‐19 era and beyond

**DOI:** 10.1002/ece3.7026

**Published:** 2020-12-15

**Authors:** Julia Chacón‐Labella, Mickey Boakye, Brian J. Enquist, William Farfan‐Rios, Ragnhild Gya, Aud H. Halbritter, Sara L. Middleton, Jonathan von Oppen, Samuel Pastor‐Ploskonka, Tanya Strydom, Vigdis Vandvik, Sonya R. Geange

**Affiliations:** ^1^ Department of Ecology and Evolutionary Biology University of Arizona Tucson AZ USA; ^2^ Department of Environmental Science Policy and Management University of California Berkeley CA USA; ^3^ The Santa Fe Institute Santa Fe NM USA; ^4^ Center for Conservation and Sustainable Development Missouri Botanical Garden St Louis MO USA; ^5^ Living Earth Collaborative Washington University St Louis MO USA; ^6^ Department of Biological Sciences University of Bergen Bergen Norway; ^7^ Bjerknes Center for Climate Research Bergen Norway; ^8^ Department of Zoology University of Oxford Oxford UK; ^9^ Section for Ecoinformatics & Biodiversity Department of Biology Aarhus University Aarhus C Denmark; ^10^ Center for Biodiversity Dynamics in a Changing World Department of Biology Aarhus University Aarhus C Denmark; ^11^ Herbario Nacional de Bolivia Universidad Mayor de San Andrés La Paz Bolivia; ^12^ Department of Ecology Environment and Plant Sciences Stockholm University Stockholm Sweden

**Keywords:** data sharing, early career, inclusivity, networking, online collaboration, skill development

## Abstract

The COVID‐19 crisis has forced researchers in Ecology to change the way we work almost overnight. Nonetheless, the pandemic has provided us with several novel components for a new way of conducting science. In this perspective piece, we summarize eight central insights that are helping us, as early career researchers, navigate the uncertainties, fears, and challenges of advancing science during the COVID‐19 pandemic. We highlight how innovative, collaborative, and often Open Science‐driven developments that have arisen from this crisis can form a blueprint for a community reinvention in academia. Our insights include personal approaches to managing our new reality, maintaining capacity to focus and resilience in our projects, and a variety of tools that facilitate remote collaboration. We also highlight how, at a community level, we can take advantage of online communication platforms for gaining accessibility to conferences and meetings, and for maintaining research networks and community engagement while promoting a more diverse and inclusive community. Overall, we are confident that these practices can support a more inclusive and kinder scientific culture for the longer term.

## Graphical abstract

We summarize eight insights learned from our own experience of collaborating internationally in the era of COVID‐19. The innovative, collaborative and Open Science responses that are currently being developed can serve as components of a new way of conducting international Science during and in a post‐pandemic world.

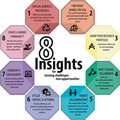

## 1 INTRODUCTION

The COVID‐19 pandemic is pushing the world into a multidimensional crisis that is disrupting global education and research (IAU, 2020; Witze, 2020; World Bank, 2020). Closed institutions and facilities, travel bans, hiring freezes, suspended meetings, a sudden shift to online teaching, visa restrictions, and limits to field campaigns are just a few of the multiple challenges that educators and researchers face. How long‐lasting and severe these impacts will be for a typically field‐ and laboratory‐based, international, and collaborative field such as Ecology is still unknown. At the same time, other well‐known issues in academia such as inequalities based on gender (Costa, 2020; Wallace & York, 2020), ethnicity and sociocultural background (Brandt et al., 2020; Dennis et al., 2005), and challenges for researchers in developing countries (Moakofhi et al., 2017; Wanelik et al., 2020) are now exacerbated as a result of the actions to prevent the spread of COVID‐19.

As the pandemic is affecting almost every aspect of our profession, the many challenges and consequences of working in academia during COVID‐19 have been thoroughly discussed (Malisch et al., 2020; Myers et al., 2020; Staniscuaski et al., 2020; Walker et al., 2020). However, the scientific community may be missing out on opportunities for strengthened collaboration and reciprocal support by not sufficiently discussing unique opportunities that have emerged for doing science during these new circumstances. The current COVID‐19 crisis provides us with a unique opportunity to gain new perspectives on how we have done science in the past—what did not work so well, what the barriers were to conducting science—and to explore what we can gain from this experience to improve our future. If “necessity is the mother of invention,” then, in the light of COVID‐19, we find ourselves with a learning‐by‐doing window of opportunity. Specifically, the innovative, collaborative, and Open Science responses that are currently being developed may help to promote a community reinvention in academia.

**Figure 1:**
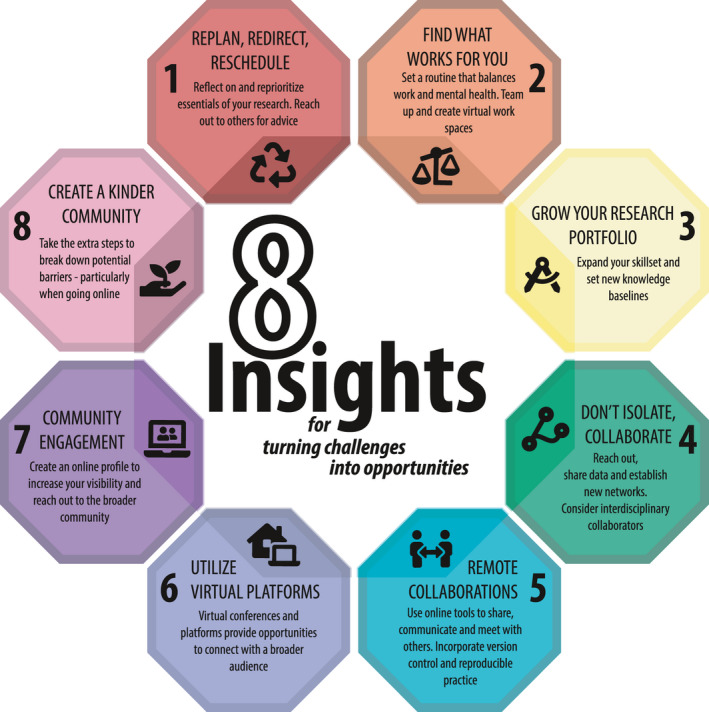
We summarize eight central insights for turning challenges into opportunities as we look to establish a new way of conducting science during and in a postpandemic world

We are a diverse group of mostly Early Career Researchers (ECRs), who met during an international postgraduate field course on plant functional traits,^1^ which was suddenly disrupted by the pandemic (see Cotner et al., 2020). This experience, in addition to adjusting to the new normality of remote‐based working once we returned home, inspired us to reflect on the creative problem‐solving approaches being taken to maintain focus, along with establishing and maintaining partnerships. Here, we summarize eight central insights learned from our own experience of doing research and collaborating internationally in the era of COVID‐19 that are helping us to continue working as ECRs in ecology during the pandemic (summarized in Figure 1). We focus on (1) how to navigate the current uncertainties, fears, and challenges of working under a new reality, and (2) how new practices for remote collaboration can serve as components of a new way of conducting science during and in a postpandemic world. We argue that even in isolation, there are multiple opportunities for doing cutting edge collaborative science and scientific outreach.

## 2 THE IMPORTANCE OF RESILIENCE: REPLAN, REDIRECT, RESCHEDULE

One of the biggest challenges of the pandemic is the inability for many to either complete field campaigns or have access to laboratory facilities and equipment. For example, losing just one year of data can be damaging for the ability of many researchers, particularly ECRs who have limited timeframes to complete their training or research projects (Inouye et al., 2018; Paula, 2020). ECRs that have to cancel their field studies or do not have access to laboratory equipment are especially vulnerable in our discipline compared to established researchers, as training and hands‐on experience are usually gained through field campaigns and laboratory work. Here, the first step toward not only maintaining research activity, but incorporating this new reality into planning for the future, is to realize that every project suffers bottlenecks that delays in its completion, and that these are often beyond our control (Bielczyk et al., 2018). A useful starting point is to invest some time to reflect on the research priorities of the project, and to determine which parts should be focused on and which can and may need to be rescheduled or changed. A first question to ask to start this thought process is “what really needs to happen this year; this field season; this upcoming week?” Maybe it is not possible to collect all the data that were planned, so prioritizing the essentials is important. Indeed, instilling a routine of constantly rehashing the central research goals, (re)prioritizing goals, and not being afraid to update or change them is an essential part of building personal resilience (McEwen, 2012).

Personal resilience is the ability to positively adapt to adversity (Fletcher et al., 2013). Building personal resilience is a key trait for researchers, and COVID‐19 has only underscored its importance. For many ECRs, rescheduling projects may seem too disappointing or may even not be an option, and having to redirect the research focus may seem challenging if not insurmountable. If project reframing is required, it is important to think carefully and draw upon the support of supervisors and colleagues, in order to balance productivity and progress in redirecting research with topics that still are of interest and maintain engagement. In such cases, initiating conversations with supervisors and colleagues can provide a different perspective on a problem. Don't be afraid to think big or to share fears—enabling mutual understanding is crucial, and especially more experienced colleagues are likely to have gone through similar situations in their careers. Discussing with peers (i.e., in a laboratory meeting) the main goals and hypotheses to be tested in the project will promote reflection on what the bigger picture was, and enable easier identification of which goals can still be accomplished. Engaging with colleagues will provide feedback from different perspectives, helping us to think outside the box, and eventually yielding new ideas.

## 3 DIFFICULTIES TO FOCUS?: THE POWER OF DOING SOMETHING EVERY DAY

While we find ourselves in these uncertain and stressful times, it can be difficult to know what we should expect from ourselves in terms of productivity and focus. Here, it is important to acknowledge that outside influences can play a big role in how we feel, especially among students and academics for whom mental health is becoming an increasing focus (Evans et al., 2018; Levecque et al., 2017; Moulin, 2020). Between the camps of “this is a time to be extremely productive” and “this is a time of great despair,” there is a lot of scope for personal and professional victories. For many of us, one of the big issues we are facing is the inability to focus. We are all adapting to how this pandemic is affecting our home and work lives (Ahmad, 2020a, 2020b). Our capacity to maintain focus during this time should be seen as a learning process and an inability to focus should not be seen as a failure. We do not know how long this crisis will last, so it is vital that, as we adjust to this “new normal,” we prioritize our own mental and physical well‐being (Ahmad, 2020b; Evans et al., 2018). This means ensuring we maintain social interactions, in‐person or virtual, engage in exercise, and do things that provide psychological relief (Barry et al., 2018).

Still, workplace responsibilities do not disappear, even if we may not be in the actual physical workplace just now. With this in mind, what strategies are available to help us, as we focus on how to ease back into work? Approaches may involve trying to do something every day, or breaking down tasks into small goals, making to‐do lists where small tasks can be ticked off, and establishing a clear daily or weekly routine. Such small and steady approaches help maintain momentum and provide a sense of accomplishment. During lockdown, a key part of developing such routines is being able to maintain the distinction between home and office. Even when working from home, one could commit to “virtual commutes” (if possible): for example, a 15‐min walk before and after work to separate the day. It is also important to take breaks—enjoy that lunch hour, or a cup of coffee. Having a designated office space, or setup, may also help maintain focus. Lastly, as we find ourselves with more online meetings, it could be advantageous to set aside meeting‐free days or afternoons, so as to reduce distractions and have blocks of time to dedicate to tasks that demand more focus.

There are also ways to work together to promote productivity and focus in laboratory or research groups, many of which also support developing strong group dynamics. One option is to establish daily morning check‐ins, where all laboratory members meet virtually for 15 min to briefly discuss how everyone is doing, outline the day's plan, and share other news. These short meetings can help set the tone for the working day, and also promote focus and productivity through peer feedback and accountability. Other possibilities could take the form of “virtual pomodoros” with colleagues, where there are 25 min of work followed by 5 min to relax and chat. By hearing and seeing other people working, we are encouraged to keep focus, and we are rewarded with opportunities for conversation and access to colleagues to quickly check ideas or new developments. An extended version of this is virtual writing retreats, which can be a productive way to focus on a single piece of work (grant proposal, manuscript, a section of code), while also maintaining social contact.

## 4 CULTIVATING YOUR RESEARCH PORTFOLIO

Early career researchers in academia often struggle to manage competing pressures. Bielczyk et al. (2018) provide useful guidelines for better self‐management. One of their key suggestions is to envision our research activity as an “investment portfolio,” diversifying the skills that we acquire so the inability to complete a project due to unforeseen events does not ruin our entire career or project. While traditionally a lot of the effort is devoted to data collection, now may be the time to circumvent the lack of access to laboratories and fields by reorganizing and prioritizing other career development skills or to revisit scientifically important tasks that are usually placed on standby. Consider expanding skill sets with participation in online courses, working on side projects, cleaning and documenting data that have been collected for years or finally finishing (and publishing) that endless paper.

An interesting option to consider when primary data collection is impeded is working on evidence synthesis, such as systematic reviews or meta‐analysis. As evidence synthesis is being more integrated into the broader research community (Nakagawa et al., 2020), opportunities arise to better prioritize future research efforts (Grainger et al., 2020). Data synthesis approaches can help in “closing the loop” of the research process that originated on a research question, by detecting when such questions have accumulated enough quality evidence and which are the research gaps that still need to be addressed (Grainger et al., 2020). For ECRs, building skills in evidence synthesis could bolster future employment opportunities and increase research impact. Training in evidence synthesis approaches could possibly replace literature reviews traditionally forming the initial chapter of many honors or doctoral theses (Méndez, 2018). Evidence synthesis is also becoming increasingly crucial in the science–policy interface, both internationally through UN‐mandated science synthesis approaches like the Intergovernmental Panel on Climate Change and the Intergovernmental Science‐Policy Platform on Biodiversity and Ecosystem Services (IPCC, IPBES), and on a more local scale through various regional and governmental mechanisms (examples: European Food Safety Authority, http://www.efsa.europa.eu/; Conservation Evidence, https://www.conservationevidence.com/).

## 5 TURN ISOLATION INTO COLLABORATION

As data collection is put on hold in many parts of the world, there is an increased opportunity to invest in open access to data, collaborative networks, and research infrastructure (Kituyi, 2020). Collaborative projects can help to improve the quality of ecological research by allowing data sharing (creating larger datasets; Pannel et al., 2019), facilitating problem solving (Goring et al., 2014), and tackling global problems (i.e., global change) while generating a more integrative understanding of ecosystems (Pannel et al., 2019). To initiate new collaborations, we need to be proactive, ask around if colleagues have data they might have had lying in the drawer for a while, seek funding to promote collaborations, or reach out to potential data sources or collaborators whose work looks interesting. Here, mentors and more senior colleagues may have an important role in facilitating networking and collaboration for ECRs (Gibson et al., 2019; Oni et al., 2016). In addition, Pannel et al. (2019) provide a list of initiatives and platforms to promote collaborations and interdisciplinary research that might help to connect ECRs. Alternatively, we should consider if our data are suitable for submitting to an existing collaborative network, or data repository. This could not only provide important opportunities for others, but also contribute to large‐scale synthesis projects (as highlighted above), which will also increase the impact of our own work in the long term (Goodman et al., 2014; Goring et al., 2014). As the pandemic has hit some countries harder than others, or has peaked at different times, there might be possibilities for someone elsewhere to replicate, or build upon, the project we had in mind, but either way, such collaborations will likely be fruitful at some point in the future.

With scientific endeavors increasingly drawing upon multidisciplinary approaches, these may open further opportunities to collaborate. Interdisciplinary research becomes especially relevant when we are facing a complex problem that can be addressed from different perspectives. In the context of the COVID‐19 crisis, many of the skills or resources available to ecological researchers could be redeployed (although we must be careful when applying ecological inference tools to other fields; Carlson et al., 2020). For example, a group of ecologists from the Biodiversity and Conservation Area at Universidad Rey Juan Carlos (Madrid, Spain) initiated an interdisciplinary project to develop a technique for COVID‐19 diagnosis, now integrating ecologists and microbiologists as well as different public hospitals in Madrid. In their innovative approach, they are using infrared spectrometry (Vis‐NIRs), a technique generally employed to identify mineral nutrients and organic compounds in plant tissues or soil samples, to develop an inexpensive and reliable new testing method (http://boscalia.org/en/covinirs‐2). Interdisciplinary projects can develop in any context and not necessarily for COVID‐19, but this example highlights that these short‐term opportunities, born out of necessity, may in the future become longer term collaborations.

## 6 REMOTE COLLABORATIONS: ONLINE PLATFORMS, VERSION CONTROL, AND REPRODUCIBILITY TOOLS

The COVID‐19 situation has shown that as researchers we can do a lot of our work and collaborate with other researchers remotely. But working remotely sometimes raises a lot of challenges in terms of “real‐time” collaboration (Geange et al., 2020; Holt et al., 2020; Trogisch et al., 2020). Online collaboration tools enable us to work together efficiently, either through facilitating communication, the sharing or storage of files and documentation, and the capacity to work in a coordinated manner (Burgio et al., 2020). Tools for online collaboration must support the three main requirements of most ecology laboratories: (1) provide opportunities for real‐time within‐team communication, (2) enable open and reproducible data management and coding workflows, and (3) facilitate file sharing and collaborative documents.

As the most popular tools for remote team communication, we may recommend a combination of a videoconferencing app (many such apps exist, and they have become very popular during social distancing), and online communication tools that allow group messaging. Together, these can work as a “virtual office,” allowing quick and easy access to information and almost instant feedback from colleagues. An additional advantage of many messaging apps includes their capacity to integrate with a wide array of other remote collaboration tools such as shared drives, videoconferencing apps, and shared online documents. Here, with online collaborative work, finding tools that support version control can be critical.

This is also a good time to build reproducible workflows that follow best‐practice standards for project and data management (Cook et al., 2001), code development, and data analysis (Cook et al., 2001; Michener, 2006; Borer et al., 2009; Strasser et al., 2018). Fortunately, excellent tools and guidelines exist to develop such reproducible workflows (see, e.g., the British Ecological Society’s (2017) Guide to Reproducible Code and references therein). In the time of COVID‐19 crisis, reproducible workflows have the added value that they also promote online collaboration in that they allow for easy sharing and updating of data and materials. Some basic practices to facilitate remote collaboration in these terms include the following: (1) establishing well‐documented workflows, covering all steps from project planning and data collection up to the final analysis output; (2) adopting transparent and reproducible practices, including clean and repeatable script‐based workflows that facilitates contributing, and/or integrating new information using version control repositories; and lastly (3) ensuring easy accessibility to data, protocols, methods, or teaching materials. Large amounts of ecological data can be available for not only collaborators, but also for the broader research field using data sharing platforms or data repositories (facilitating their use of data in synthesis; Halbritter et al., 2020). Establishing these practices as habits early on can help ECRs to streamline their workflows and to interact with remote collaborators and supervisors efficiently.

## 7 TAKE ADVANTAGE OF ONLINE CONFERENCE PLATFORMS AND VIRTUAL MEETINGS

The halting of many conferences due to the COVID‐19 pandemic has forced the scientific community to explore online alternatives (Achakulvisut et al., 2020; Holt et al., 2020; Lortie, 2020). Although virtual conferences cannot replace in‐person ones, and lack some of the face‐to‐face benefits (Vekkaila et al., 2018), they do hold many opportunities that could be explored by ECRs and the broader scientific community. For example, reduced attendance fees and zero travel costs may enable greater access by students, or encourage researchers to explore conferences they might not otherwise consider attending. Furthermore, virtual conferences reduce VISA hassles for participants, which may often be more prohibitive for those from developing nations and, along with financial aspects, creates larger gaps between researchers from developing and developed countries (Bradley et al., 2020). Online conferences and virtual meetings also contribute to lowering researchers’ carbon footprint (Klöwer et al., 2020). Indeed, the format and content of online conferences is changing and developing fast as a response to the pandemic. The new online format is allowing useful and impactful interactions that enable better learning in some aspects than in‐person meetings (Lortie, 2020).

Another possible benefit of a shift toward online communication platforms is an increased opportunity for access to resources and networked communities, for example, via virtual meeting spaces. Departmental seminars are a key way in which scientific insights are shared among the broader researcher community, and also provide excellent networking opportunities, particularly for ECRs. However, in the past, the capacity for institutes to invite and host guest seminar speakers has been primarily limited to those with bigger budgets. A shift toward online presentations would not only allow greater visibility for the presenter, but also enable increased access for departments/institutions where this may have been either (a) too expensive, or (b) locationally difficult, that is, many southern hemisphere countries. In a similar vein, scientific workshops and resources can also be adopted, with many traditional face‐to‐face workshops already being shifted, or supported by online platforms.

## 8 RESEARCH NETWORKS AND COMMUNITY ENGAGEMENT

In‐person meetings with informal discussions remain the main way to foster collaborations and network, especially for ECRs (Pannell et al., 2019). However, during the past decade, academics have increasingly utilized both mainstream and academic‐focused social networking sites to supplement in‐person activities such as sharing research and networking (Jordan & Weller, 2018). These platforms dissolve geographic borders, allowing researchers from across the world to share resources and engage with a variety of audiences outside their immediate academic institutions (Cheplygina et al., 2020). ECRs can improve their visibility and searchability by highlighting their research, skills, accomplishments, and publications through curating an online profile (Nentwich & König, 2014; Tachibana, 2014). This could be via personal websites, or through registering on research platforms (e.g., ORCID, Google Scholar, ResearchGate). Many researchers also engage via social media, such as microblogging sites, where they share research, network and engage in scientific discussions (Bista, 2015; Cheplygina et al., 2020). These can be a useful way to keep up to date with recent scientific developments and opportunities, with many journals and individuals promoting new insights. The rise of “altmetrics” in academia, along with both online profiles and social media platforms, plays an important role in increasing the scientific visibility of research (Sugimoto, Work, Larivière, & Haustein, 2017).

Online tools also provide a resource for ECRs to build networks outside their own research community and connect with wider society. Initiatives like Skype a Scientist and Live with Scientists enable researchers to engage in informal discussions about their research (usually in the form of a question and answer session) with different members of the community (e.g., school children or adults who do not formally engage in science). Such events can make important contributions to increase scientific literacy among nonscientists and raise awareness of current research issues. As these events are hosted virtually, ECRs can connect with communities from around the world and participants have the possibility to be exposed to a wide range of scientific ideas that may have previously been inaccessible to them.

## 9 PROMOTE A KINDER, MORE INCLUSIVE, AND DIVERSE COMMUNITY

In times of crisis, underrepresented groups are those that disproportionately suffer the consequences of a sudden change (Bapuji et al., 2020). Even before the COVID‐19 yielded the current international crisis, diversity, equality, and inclusion were already important issues with critical implications in STEM (Brower, 2020; Cech & Blair‐Loy, 2019). The systemic disadvantage of minorities and women in academic and scientific enterprises is now magnified, and potential long‐term effects of the current crisis over these underrepresented groups can be daunting (Brower, 2020; Staniscuaski et al., 2020). In the last months, we have seen a large number of reports highlighting how the pandemic is disproportionately impacting academic mothers (Staniscuaski et al., 2020; Viglione, 2020) and people or regions with low incomes or limited access to technologies (Lee et al., 2019; The Economist Intelligence Unit, 2020).

As ECRs, we need the COVID‐19 crisis to be a wakeup call for institutions and agencies to develop more flexible family‐friendly policies and action to mitigate parenthood, but especially motherhood penalties (Cech & Blair‐Loy, 2019). Such policies will help to mitigate both the exit of trained professionals from the STEM workforce and the strong difficulties that these professionals face balancing childcare or caregiver duties alongside their job responsibilities. In this context, organizational policies, the lack of work–life programs and flexible work arrangements, and increased workloads are significantly associated with the prevalence of mental health problems in academia (Levecque et al., 2017). Now more than ever it is essential to understand that being locked at home does not necessarily translate into a boost in productivity (Paula, 2020). We are all living stressful times and operating with more responsibilities and concerns than ever before: that is, family losses, sick relatives, extreme loneliness, or visa suspensions among others, but are also finding themselves with fewer resources to complete their work. Now is the opportunity to use this time to develop a more empathic and supportive community by adjusting our expectations of workplace productivity of both ourselves and others. Here, institutions and policymakers have a leading role in facilitating management of work–life balance and workloads (Levecque et al., 2017) and developing programs that do not compromise ECRs future job (i.e., considering extending contracts; Paula, 2020). At a personal or laboratory group level, it may be addressed by developing a value statement or code of conduct, and working toward improvements of supervisor's leadership style and team decision‐making culture within the laboratories (Fernandez & Shaw, 2020; Levecque et al., 2017).

For building a more inclusive and diverse scientific community, we must also consider that most of the new approaches for working and collaborating remotely rely upon Internet access, visual displays, and text‐based chat interactions. In this context, it is important to acknowledge the difficulties to ensure access to meetings, resources, and digital networks for everyone. For example, it is crucial to appreciate which additional challenges may be faced either by colleagues from developing countries with unequal access to technologies (Moakofhi et al., 2017), those from across different time‐zones (Andersson, 2008), or members of the scientific community with disabilities. A few simple suggestions when Internet and technology access are limited include replacing complicated online platforms for sharing materials with those that are easier to navigate and that require less data (such as email), and audio‐visual platforms by more handy and less data usage audio‐visual apps that can be accessed via mobile phones (which most people have). Another simple suggestion to take advantage of audio‐visual platforms options is to record and save meetings or lectures for later use. Ensuring flexibility with virtual meetings, allowing the coexistence of live meetings with prerecorded ones and integrating additional tools/resources for members with disabilities, is important to make sure that traditional in‐person meeting barriers (Sohn, 2019) are not transferred to virtual meetings if we are to be truly inclusive.

## 10 CONCLUSIONS: AN OPPORTUNITY FOR COMMUNITY REINVENTION IN ACADEMIA

The pandemic has illuminated that the way we do science is changing. Indeed, we are now in a process of adapting to this new reality. During this transition, each of us first need to take care of our mental and physical health, we need to help each other, and adopt new practices to maintain our capacity to focus and to promote resilience in our research practice. Within a very short time, we have shifted from a 100 percent in‐person model to an almost completely remote one. However, in this process we have also adopted new practices that hold large potential to serve as a foundation for a more international, collaborative, and Open Science model underpinned by technological developments. The question here is: “What do we want to keep for the future, and how?” The COVID‐19 crisis presents the opportunity for researchers and institutions to transition to a new hybrid model that integrates remote and in‐person workflows, taking advantage of the new skills that have recently been developed. It also presents us multiple opportunities to address long‐lasting issues in academia, such as the lack of diversity, equity and inclusivity. To take the final steps and use the momentum, we not only individually need to take action, but need the commitment and support of our institutions and research agencies. If we succeed to transform this new workstyle into routines, it could serve as a model for a community reinvention in academia. We should not miss the opportunity to take many of the eight insights for conducting science as a foundation to design the community that we want to work in.

## CONFLICT OF INTERESTS

The authors declare no competing interests.

## AUTHORSHIP CONTRIBUTIONS

**Conceptualization:** JCL and SRG lead; MB, BJE, WFR, RG, AHH, SLM, JvO, SPP, TS equal. **Writing ‐ Original Draft Preparation:** JCL and SRG lead; MB, BJE, WFR, RG, AHH, SLM, JvO, SPP, and TS equal. **Writing ‐ Review and Editing:** JCL and SRG lead; MB, BJE, WFR, RG, AHH, SLM, JvO, SPP, TS, and VV equal. **Visualization:** TS lead; MB, JCL, BJE, WFR, SRG, RG, AHH, SLM, JvO, SPP, TS, and VV equal.

## ENDNOTE

^1^
https://plantfunctionaltraitscourses.w.uib.no/
